# Modeling based on machine learning to investigate flue gas desulfurization performance by calcium silicate absorbent in a sand bed reactor

**DOI:** 10.1038/s41598-024-51586-7

**Published:** 2024-01-10

**Authors:** Kamyar Naderi, Mohammad Sadegh Kalami Yazdi, Hanieh Jafarabadi, Fatemeh Bahmanzadegan, Ahad Ghaemi, Mohammad Reza Mosavi

**Affiliations:** 1 School of Chemical, Petroleum and Gas Engineering, Iran University of Science and Technology, Narmak, Tehran, 16846 Iran; 2 Department of Electrical Engineering, Iran University of Science and Technology, Narmak, Tehran, 16846-13114 Iran

**Keywords:** Environmental chemistry, Chemical engineering

## Abstract

Flue gas desulfurization (FGD) is a critical process for reducing sulfur dioxide (SO_2_) emissions from industrial sources, particularly power plants. This research uses calcium silicate absorbent in combination with machine learning (ML) to predict SO_2_ concentration within an FGD process. The collected dataset encompasses four input parameters, specifically relative humidity, absorbent weight, temperature, and time, and incorporates one output parameter, which pertains to the concentration of SO_2_. Six ML models were developed to estimate the output parameters. Statistical metrics such as the coefficient of determination (R^2^) and mean squared error (MSE) were employed to identify the most suitable model and assess its fitting effectiveness. The random forest (RF) model emerged as the top-performing model, boasting an R^2^ of 0.9902 and an MSE of 0.0008. The model's predictions aligned closely with experimental results, confirming its high accuracy. The most suitable hyperparameter values for RF model were found to be 74 for n_estimators, 41 for max_depth, false for bootstrap, sqrt for max_features, 1 for min_samples_leaf, absolute_error for criterion, and 3 for min_samples_split. Three-dimensional surface plots were generated to explore the impact of input variables on SO_2_ concentration. Global sensitivity analysis (GSA) revealed absorbent weight and time significantly influence SO_2_ concentration. The integration of ML into FGD modeling offers a novel approach to optimizing the efficiency and effectiveness of this environmentally crucial process.

## Introduction

Sulfur dioxide (SO_2_) is a prominent atmospheric contaminant that plays a substantial role in the degradation of air quality. This pollutant notably influences the natural environment and the global climate system^1,2^. Industrial processes, especially those involving fossil fuel combustion, are recognized as significant sources of SO_2_ emissions^3^. Power plants and industries contribute to over 70% of the total anthropogenic SO_2_ emissions, making them the primary contributors to this environmental concern^4^. Various technologies have been developed to mitigate SO_2_ emissions, such as fuel switching^5^, catalytic converters^6^, coal preparation^7^, low-sulfur fuels^8^, boiler modernization^9^, fluidized bed combustion^10^, and flue gas desulfurization^11^. When choosing a method for removing or lowering the emission of SO_2_ from flue gases, it is necessary to consider a range of criteria. The ideal approach should encompass safety, environmental sustainability, and cost-effectiveness while minimizing potential losses and eliminating the issue of fouling^12^.

Flue gas desulfurization (FGD) is one of the most effective emission control technologies used in power plants, and it plays a pivotal role in reducing SO_2_ emissions^13^. Several FGD systems have been developed, and the selection process involves considering technical factors and making an economic decision. Notable concerns encompass the extent of desulfurization achievable by the technique and its adaptability. The majority of FGD systems employ an alkali sorbent, such as limestone (calcium carbonate), quicklime (calcium oxide), hydrated lime (calcium hydroxide), or occasionally sodium and magnesium carbonate and ammonia, to trap the acidic sulfur compounds present in the flue gas. Regardless of the circumstances, the alkalis chemically interact with SO_2_ in the presence of water (such as a mist of slurry containing the sorbent) to generate a combination of sulfite and sulfate salts. This reaction might occur either inside the entire solution or on the moistened surface of the solid alkali particles^14^. FGD technologies are frequently categorized into wet, semi-dry, or dry processes^15^.

The ADVACATE process was created as an alternative way to clean the flue gas in coal-fired power plants by duct injection. It offers a smaller physical size and lower initial cost than wet desulfurization systems, making it a practical option for upgrading existing plants to meet stricter flue gas cleaning standards^16^. The ADVACATE process involves the introduction of ADVACATE solids into the cool-side duct to mitigate the presence of SO_2_, NO_x_, and several other pollutants within the flue gas. The removal process occurs in the gas duct, and the bag filter particle control device exhibits greater significance. Solid ADVACATE materials are formed through the chemical reaction between hydrated lime and recycled fly ash derived from power plants. The chemical as mentioned above process results in the formation of a calcium silicate hydrate solid with a significant degree of porosity, enabling it to retain a considerable quantity of water (~ 50 wt.%) while maintaining the handling characteristics of a powder, as shown by Eqs. 1–3. A substantial quantity of water and alkalinity facilitates the elimination of acid gases and the efficient conversion of solids^17^.

1 $${\text{Ca}}{({\text{OH}})}_{2}\leftrightarrow {{\text{Ca}}}^{2+}+2{({\text{OH}})}^{-}$$

2 $${({{\text{SiO}}}_{2})}_{x}+2{{\text{H}}}_{2}{\text{O}}+{({\text{OH}})}^{-}\leftrightarrow {({{\text{SiO}}}_{2})}_{x-1}+{\text{Si}}{({\text{OH}})}_{5}^{-}$$

3 $${{\text{Ca}}}^{2+}+y{\text{Si}}{({\text{OH}})}_{5}^{-}+\left(2-y\right){\left({\text{OH}}\right)}^{-}+(z-2y-1){{\text{H}}}_{2}{\text{O}}\to ({\text{CaO}}) {\left({{\text{SiO}}}_{2}\right)}_{y}{{({\text{H}}}_{2}{\text{O}}) }_{z}$$

**Figure 1:**
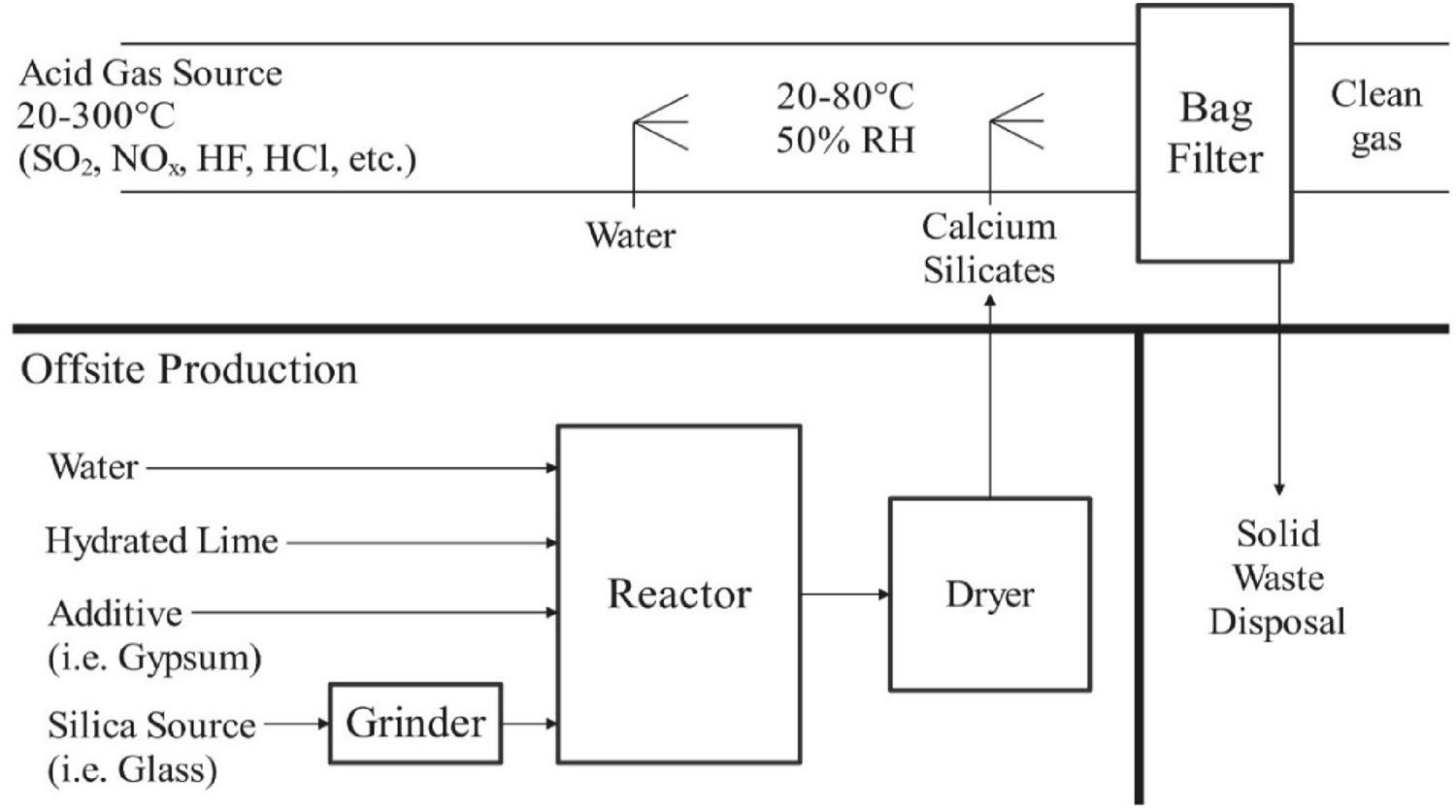
Small source ADVACATE process.

Figure 1 depicts the stages of preparation. Depending on the size of the starting silica particles, the first step is grinding. The silica undergoes a high-temperature reaction with lime and other additions in an aqueous medium. After the sludge has been dewatered and dried, it can be sent to the source sites. The gas–solid contact can be achieved using a duct-injection/baghouse filter configuration. The gas can also be utilized as a filter medium in a fixed bed medium.

The FGD process has shown promising potential for efficient SO_2_ removal. Dzhonova et al.^18^ studied the Wellman-Lord method for removing SO_2_ from flue gases in combustion systems. The method uses sodium sulfite to absorb SO_2_ and produce sodium bisulfite. The regenerated solution can be reused in the absorber. The authors found the method more cost-effective than other FGD methods and suggested techniques to enhance it. They introduced a new technology with lower steam consumption, heat utilization for heating district heating water, and lower capital costs. The study by Özyuğuran and Meriçboyu^19^ compared the desulfurization efficiencies of hydrated lime and dolomite absorbents from flue gases. They subjected them to sulfation at 338K and measured their weight increase during the SO_2_ reaction. The researchers found that the total sulfation capacities increased with increased surface areas and decreased mean pore radius, indicating that the physical properties of absorbents significantly influence their sulfation properties. A study developed by Xu et al.^20^ integrated the FGD-CABR system to remove NO_x_ and SO_2_ from flue gas, achieving 100% removal efficiency. The primary sulfur compound was sulfide, with the spray scrubber partially facilitating NO_x_ removal through sulfide-oxidizing and nitrate-reducing bacteria enrichment. Most NO_x_ was converted into harmless N_2_ in the expanded granular sludge bed reactor. Stanienda-Pilecki^21^ explored the use of limestone sorbents with increased magnesium content in FGD processes in power stations. Triassic limestones in Poland, consisting of low magnesium calcite, high magnesium calcite, dolomite, and huntite, have various magnesium contents. The increased magnesium content in the sorbent positively impacted the dry method of desulfurization, especially when using fluidized bed reactors. Because magnesium ions are unstable, they made it easier to remove carbon from carbonate phases at temperatures similar to those used to remove carbon from dolomite. This results in a faster and more effective desulfurization process.

Over the past few years, numerous methods have been proposed to predict SO_2_ and other emissions from power plants. Among these approaches, mathematical models, and machine learning (ML) models have generated significant scientific interest. However, accurately modeling the concentration of SO_2_ is a challenging task mathematically. Some studies simplify this system by incorporating assumptions, leading to errors in predictions. Furthermore, the calculations utilized in these mathematical models require substantial computing resources^22^. ML approaches are extensively considered due to their accuracy, fast speed, and capability to do nonlinear calculations, diagnosis, and learning. Additionally, recent advancements in predictive modeling techniques, such as adaptive sampling based surrogate modeling, have gained popularity ^23^. So far, extensive studies have been carried out in the field of FGD by ML approach. Zhu et al. ^24^ developed a highly effective ML approach for estimating SO_2_ absorption capacity in deep eutectic solvents (DESs). Based on critical parameters like molecular weight, water content, pressure, and temperature, the model was the most accurate in forecasting 480 DES-SO_2_ phase equilibria, ensuring its dependability and generalizability. Grimaccia et al.’s ^25^ study aimed to create a model for a proprietary SO_2_ removal technology at the Eni oil and gas treatment plant in southern Italy. The goal was to develop an ML algorithm for unit description, independent of the licensor and more flexible. The model used ANNs to predict three targets: SO_2_ flow rate to the Claus unit, SO_2_ emissions, and steam flow rate to the regenerator reboiler. The data-driven technique accurately predicted targets, allowing optimal control strategies and plant productivity maximization. Xie et al.^26^ introduced a long short-term memory (LSTM) neural network to improve the WFGD process in thermal power plants. The model achieved a high prediction accuracy of 97.7%, surpassing other models. The modified LSTM model was rigorously tested and validated, demonstrating good prediction effect and high stability. Yu et al.^27^ developed a dynamic model to predict SO_2_-NO_x_ emission concentration in fluidized bed units, aiming to meet emission standards and create an environmentally friendly pollutant removal mode. The model used Pearson coefficients, an extreme learning machine, and a quantum genetic algorithm to optimize connection weights, accurately imitating actual data trends. Yin et al.^28^ developed a hybrid deep learning model integrating a convolutional neural network (CNN) and LSTM to improve the accuracy of predicting SO_2_ emissions and removal in limestone-gypsum WFGD systems. The model captures local and global dynamics and temporal characteristics and introduces an attention mechanism (AM) to allocate weights to the outlet SO_2_ sequence at different time points. The model outperforms alternative methodologies in predictive accuracy. Makomere et al.'s^29^ research examined the effectiveness of ANN in modeling desulfurization reactions using Bayesian regularization and Levenberg–Marquardt training algorithms. The shrinking core model was used, revealing the chemical reaction as the rate-controlling step. Bayesian regularization was preferred due to its flexibility and overfitting minimization capabilities. The hyperbolic tangent activation function showed the best forecasting ability. An investigation by Uddin et al.^30^ on the limestone-forced oxidation (LSFO) FGD system in a supercritical coal-fired power plant. Monte Carlo experiments showed that optimal operation could reduce SO_2_ emissions by 35% at initial concentrations of 1500 mg/m^3^ and 24% at initial 1800 mg/m^3^ concentrations. These findings were crucial for reducing emissions in coal power plants and developing effective operational strategies for the LSFO FGD system. Fedorchenko et al.^22^ presented an optimization strategy for FGD using data mining. A modified genetic method based on ANNs was developed, allowing for better prediction of time series characteristics and efficiency. The method used adaptive mutation, allowing less important genes to mutate more likely than high suitability genes. Comparing this method with other methods, the new method showed the smallest predictive error and reduced prediction time, thereby increasing efficiency and reducing SO_2_ emissions. Adams et al.^31^ developed a deep neural network (DNN) and least squares support vector machine (LSSVM) to predict SO_x_ and NO_x_ emissions from coal conversion in energy production. The models were trained on commercial plant data and examined the impact of dynamic coal and limestone properties on prediction accuracy. The results show that training without assumptions improved testing accuracy by 10% and 40%, respectively. Interactive and pairwise correlation features reduced computational time by 46.67% for NO_x_ emission prediction. A summary of the studies conducted in the field of ML for FGD and their results are given in Table 1.

**Table 1:** A summary of some studies used ML to model FGD.

Input variables	Type of sorbent	Number of datasets	ML models	Evaluation measures							References
				R^2^	MSE	RMSE	nRMSE	MAE	EMAE (%)	MAPE (%)	
Molecular weight, water content of DES, pressure, and temperature	DES	480	MLP (Levenberg–Marquardt)	0.979	0.001	–	–	–	–	4.76	^24^
			CFF (Levenberg–Marquardt)	0.979	0.001	–	–	–	–	4.40	
			RBF	0.809	0.009	–	–	–	–	14.79	
			RNN (Scaled Conjugate Gradient)	0.909	0.005	–	–	–	–	9.36	
			CFF (Bayesian Regularization)	0.988	0.001	–	–	–	–	5.73	
			ELM (Whale optimization algorithm)	0.926	0.008	–	–	–	–	20.87	
			RBF (Whale optimization algorithm)	0.934	0.007	–	–	–	–	18.44	
Concentration, mass flow, temperature, pressure, level	A proprietary solvent	35	MLP (Levenberg–Marquardt)	0.981	–	2.06	0.031	–	8.62	–	^25^
Slurry pH, slurry flow, slurry density, temperature	Slurry	22,000	LSTM	0.977	0.006	–	–	–	–	–	^26^
Limestone flow, coal flow rate, unit load, total airflow, primary airflow, secondary airflow, oxygen measuring point A, oxygen measuring point B	Lime	16,000	Genetic algorithm	0.942	–	161.697	–	408.183	–	6.6	^27^
			MLP	–	–	2.901	–	2.325	–	–	^28^
			SVM	–	–	3.123	–	2.380	–	–	
Power, the inlet SO_2_ concentration of flue gas in the booster fan, the inlet temperature of the original flue gas entering the booster fan, input motor current of circulation pump C, pH value of the limestone slurry, input motor current of circulation pump B, original flue gas flow, motor current of oxidation fan A, converted output motor current of circulation pump C	Limestone	4320	LSTM	–	–	1.919	–	1.536	–	–	
			CNN-LSTM	–	–	1.603	–	1.208	–	–	
			CNN-LSTM-AM	–	–	1.436	–	0.973	–	–	
Diatomite/Ca(OH)_2_, hydration time, hydration temperature, inlet SO_2_, sulfation temperature	Diatomite/ Hydrated lime	50	MLP (Levenberg–Marquardt)	0.985	0.134	–	–	–	–	–	^29^
			MLP (Bayesian Regularization)	0.997	0.023	–	–	–	–	–	
pH, inlet SO_2_, inlet temperature, inlet NO_x_, inlet O_2_, oxidation air, absorber slurry density, inlet humidity, inlet dust	Slurry	65,000	MLP	0.8295	–	–	–	–	–	–	^30^
			Linear regression	–	9103	–	–	79.24	–	–	^22^
			Polynomial regression	–	9031	–	–	77.84	–	–	
			Logistic regression	–	9041	–	–	78.56	–	–	
			Nearest neighbour algorithm	–	7421	–	–	75.76	–	–	
SO_2_ inlet, water consumption in an absorber, lime consumption, secondary reagent consumption	Lime	5330	RF		7201	–	–	74.15	–	–	
			Ant colony optimization algorithm	–	7523	–	–	73.89	–	–	
			MLP with one hidden layer	–	6785	–	–	59.85	–	–	
			MLP with two hidden layers	–	5795	–	–	40.98	–	–	
			MLP with two hidden layers and the developed genetic algorithm	–	3253	–	–	24.95	–	–	
Boiler capacity, fuel spoon air flow rate, fuel feeding front flow rate, combustion airflow rate, primary airflow rate, primary air temperature, upper secondary airflow rate, lower secondary airflow rate, secondary air temperature, bed pressure, Furnace temperature	Limestone	24,000	DNN	0.9380	–	2.304	–	–	–	–	^31^
			LSSVM	0.9525	–	2.014	–	–	–	–	

Considering the prevailing research landscape focused on traditional modeling approaches in the realm of FGD, this study strategically addresses critical research gaps. Specifically, our work pioneers the application of ML techniques to model and predict the performance of calcium silicate absorbents within the context of a sand bed reactor. Additionally, using ML in sand bed reactors in FGD is a new idea that goes against traditional ways of doing things and shows how advanced modeling techniques can be used to get the best results in this reactor. This study, therefore, endeavors to fill existing research gaps and advance the state of knowledge in the field. The study used data from experiments on FGD with a calcium silicate absorbent in a sand bed reactor as both input and output for the ML method. This research aims to utilize ML models to estimate the concentration of SO_2_ accurately and quickly in flue gas. For implementing the proposed models, 323 experimental data points collected from this work were considered. A statistical evaluation and comparison of the accuracy of the constructed ML models was conducted based on the coefficient of determination (R^2^) and mean squared error (MSE), and the best model was chosen. The results of this study can be used in power plants, environmental regulations, engineering and design, research, and development in the future.

## Theoretical background

### Setup description

**Figure 2:**
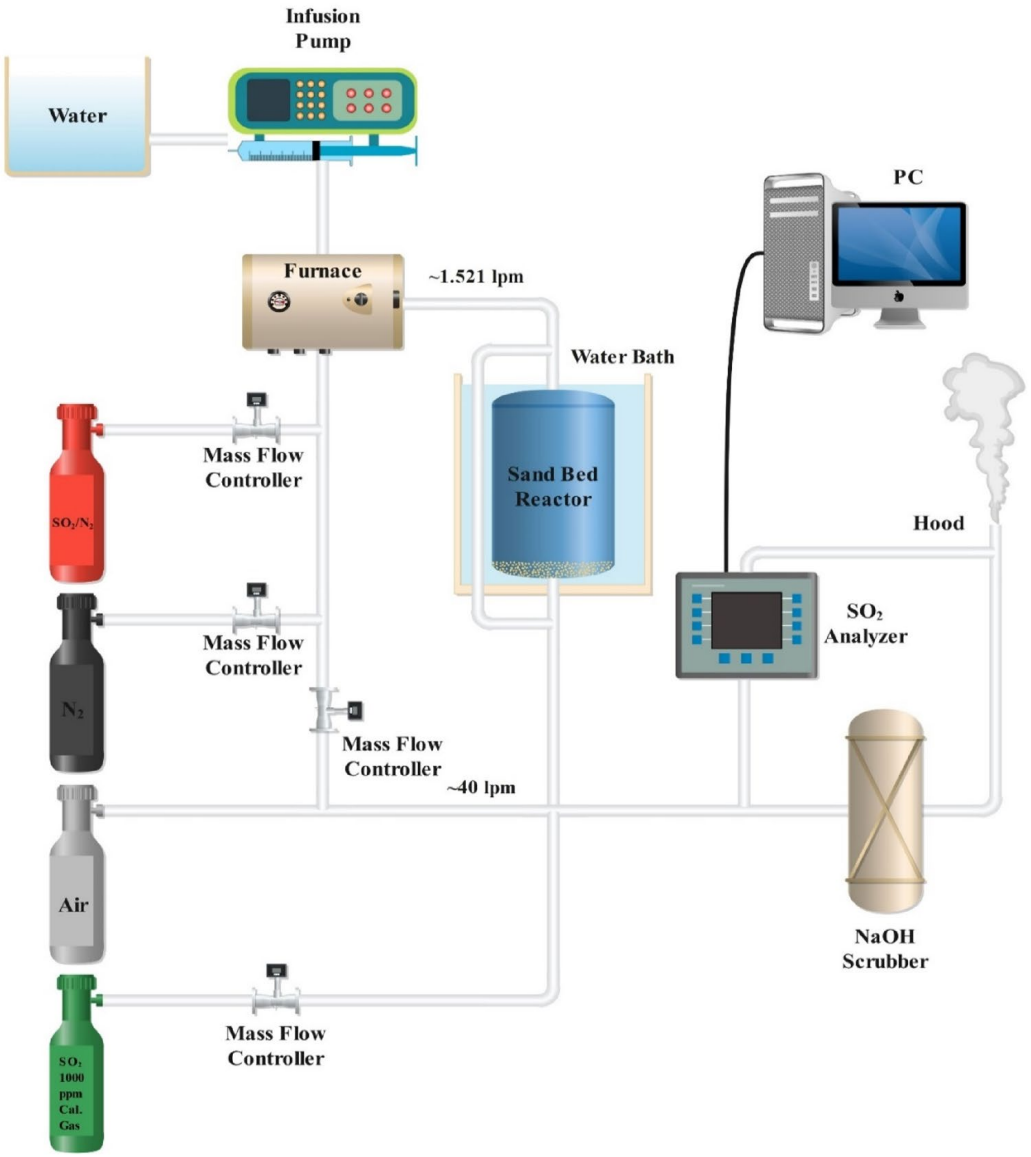
A schematic of the sand bed reactor system.

The reaction between SO_2_ and solid absorbents was studied in Arthur's sand bed reactor system^17^ and shown in Fig. 2. Compressed SO_2_/N_2_ (~ 0.5%) was diluted with either nitrogen or air, depending on the desired oxygen content, to create a simplified flue gas. The flow rates of all gases were controlled using mass flow meters and a controller box. Water was supplied to a helical Pyrex evaporator through an Infusion Pump, which humidified the flue gas. The temperature in the furnace was regulated using a voltage controller. The flow rate of water from the syringe pump was measured by monitoring the weight of the water output over time. The sand bed reactor used in the experiment was made of glass and had dimensions of 7.5 inches in length and 1.5 inches in diameter. A 2-mm coarse glass frit was placed at the bottom of the reactor to support the mixture of sand and absorbent. The reactor was sealed using a ground glass fitting secured with a metal clamp and rubber bands. It was positioned upright in a water bath, which was temperature-controlled using a dedicated controller. The concentration of SO_2_ was measured using an SO_2_ analyzer, and the output from the analyzer was automatically collected using a digitizer and PC for data analysis. A bypass line was incorporated within the temperature-controlled water bath to establish a stable operational state for the synthesized flue gas and the analytical system before the onset of the chemical reaction. The flue gas, characterized by concentrations spanning from 0 to 2000 parts per million (ppm), underwent substantial dilution with ambient air from the facility to attain concentrations within the 0 to 50 ppm range, a requisite for the analyzer. This dilution process concurrently addressed issues related to gas condensation within the analytical system by reducing the relative humidity of the gas. The predominant portion of the effluent gas stream was directed through a sodium hydroxide (NaOH) scrubbing system, which typically operated under a pH level of 13. A small vacuum pump integrated into the SO_2_ analyzer extracted a small portion of the gas.

### Data collection

**Figure 3:**
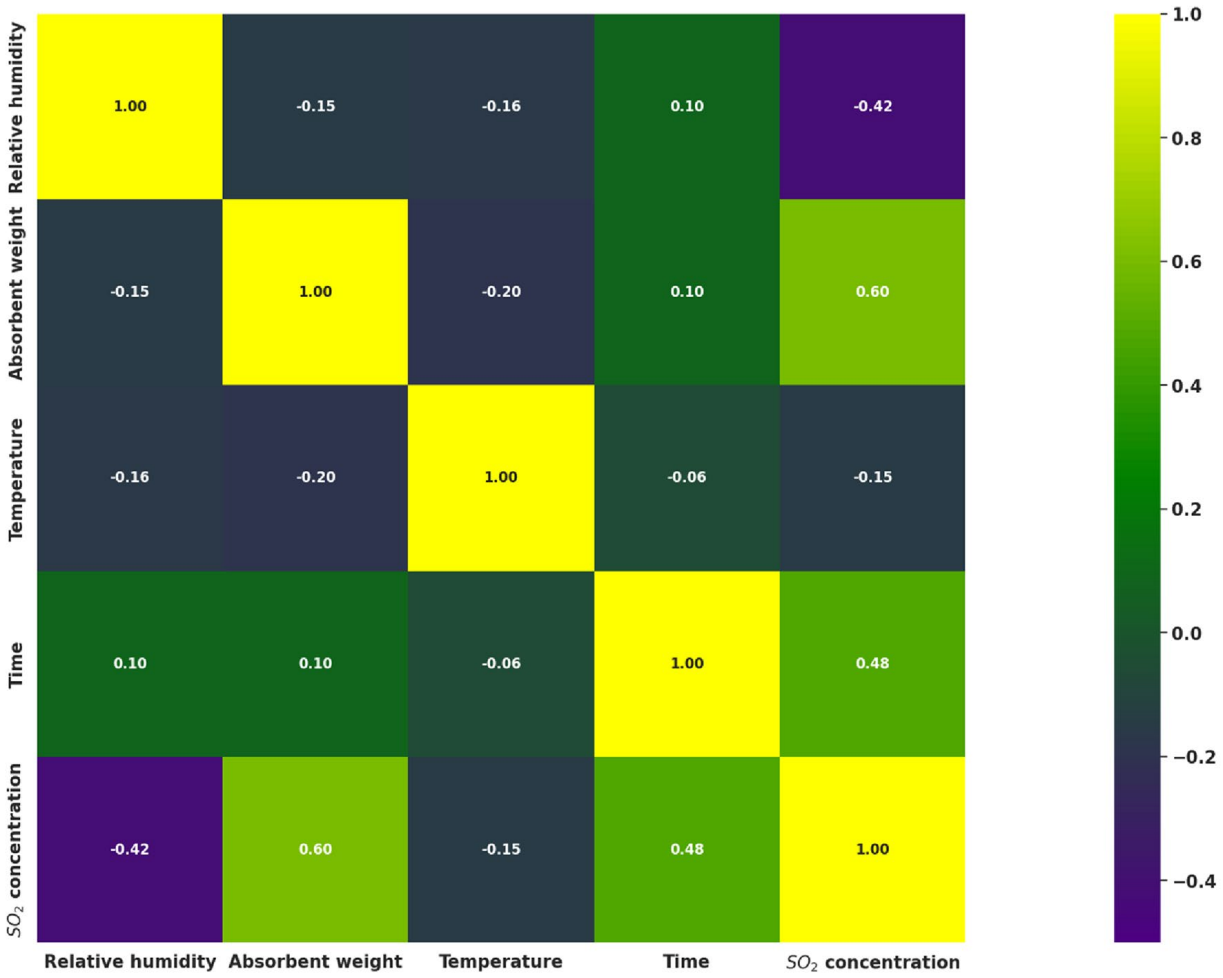
Pearson correlation matrix between each variable.

Since the concentration of SO_2_ can be affected by different operating conditions, there is a need to investigate the relationship between the outlet concentration and the parameters affecting the outlet concentration. Relative humidity, absorbent weight, temperature, and time play an essential role in the concentration of SO_2_. Therefore, relative humidity, absorbent weight, temperature, and time were included among the input variables. The SO_2_ concentration was also considered as output. Hence, this study incorporates the input variables of maximum level (max), minimum level (min), average level (mean), and standard deviation (STD), as presented in Table 2. The training and testing data for the models were acquired from Arthur^17^, yielding a dataset comprising 323 data points. The Pearson correlation coefficient matrix is the covariance of the two mentioned features and the product of their standard deviation. The correlation among the selected variables is analyzed and presented in the heatmap in Fig. 3.

**Table 2:** Statistical properties of the variables.

Variables	70% Train–20% Validation–10% test			
	Min	Max	Mean	STD
Relative humidity (%)	0	88	48.421	28.023
Absorbent weight (g)	0.025	0.1	0.087	0.028
Temperature (˚C)	36.5	50	47.84	4.949
Time (min)	1	60	22.51	13.968
SO_2_ concentration (ppm)	35.6	1036.9	808.961	266.975

### Model selection

**Figure 4:**
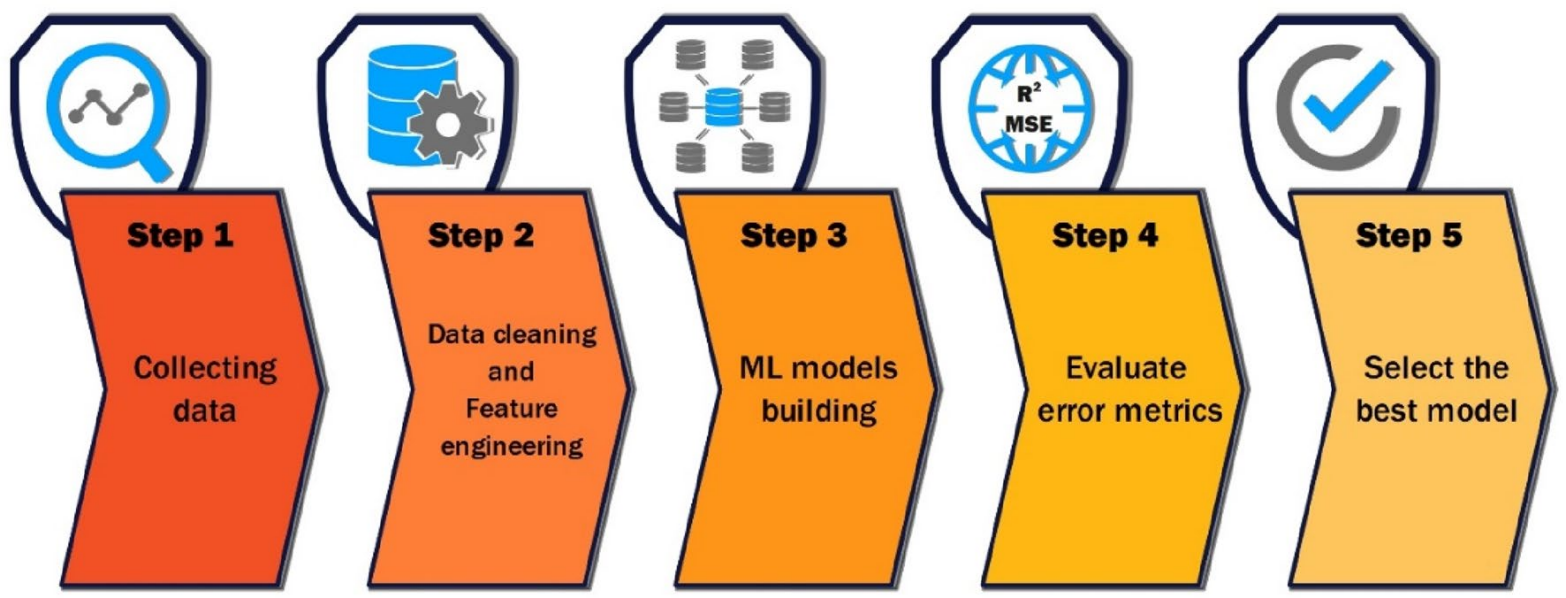
Procedure of the current ML-based modeling.

In this study, all ML analyses were conducted using the Python programming language. Various ML methods and models are available to solve clustering, classification, and regression problems. However, the challenge lies in determining which model and combination of hyperparameters would work best for a specific dataset. The optimization algorithm in this case, involves multiple learning algorithms (models) and hyperparameters. It is necessary to explore numerous combinations to maximize predictive accuracy and find the optimal set of hyperparameters. In this study, six models are used: artificial neural network (ANN), multilayer perceptron (MLP), radial basis function neural network (RBFNN), random forest (RF), extra trees regression (ETR), and support vector regression (SVR). The procedure to reach the best ML model is shown in Fig. 4.

#### Artificial neural network

An ANN is a computational model inspired by the workings of the human brain. It comprises many individual units, like artificial neurons, which are connected by coefficients known as weights. These weights together form the network structure and enable it to process information. Each of these processing units, often called processing elements (PE), has inputs with different weights, a transfer function, and produces a single output. Think of PE as an equation that balances its inputs and outputs. ANNs are often called connectionist models because the connection weights effectively serve as the network's memory^32^. While a single neuron can handle simple information-processing tasks, the true power of neural computation comes to light when these neurons are interconnected within a network. Whether ANNs possess accurate intelligence remains a topic of debate. Notably, ANNs typically consist of only a few hundred to a few thousand PEs, whereas the human brain contains about 100 billion neurons. So, artificial networks with the complexity of the human brain are still far beyond our current computational capabilities. The human brain is much more intricate, and many intellectual functions remain unknown. However, ANNs excel at processing large amounts of data and can make surprisingly accurate predictions. Nonetheless, they do not possess the kind of intelligence that humans do. Therefore, it might be more appropriate to refer to them as examples of computer intelligence. In the field of neural networks, various types of networks have been developed over time, and new ones continue to emerge regularly. However, they can all be categorized based on the functions of their neurons, the rules they use to learn, and the formulas governing their connections^33^.

#### Multi-layer perceptron

**Figure 5:**
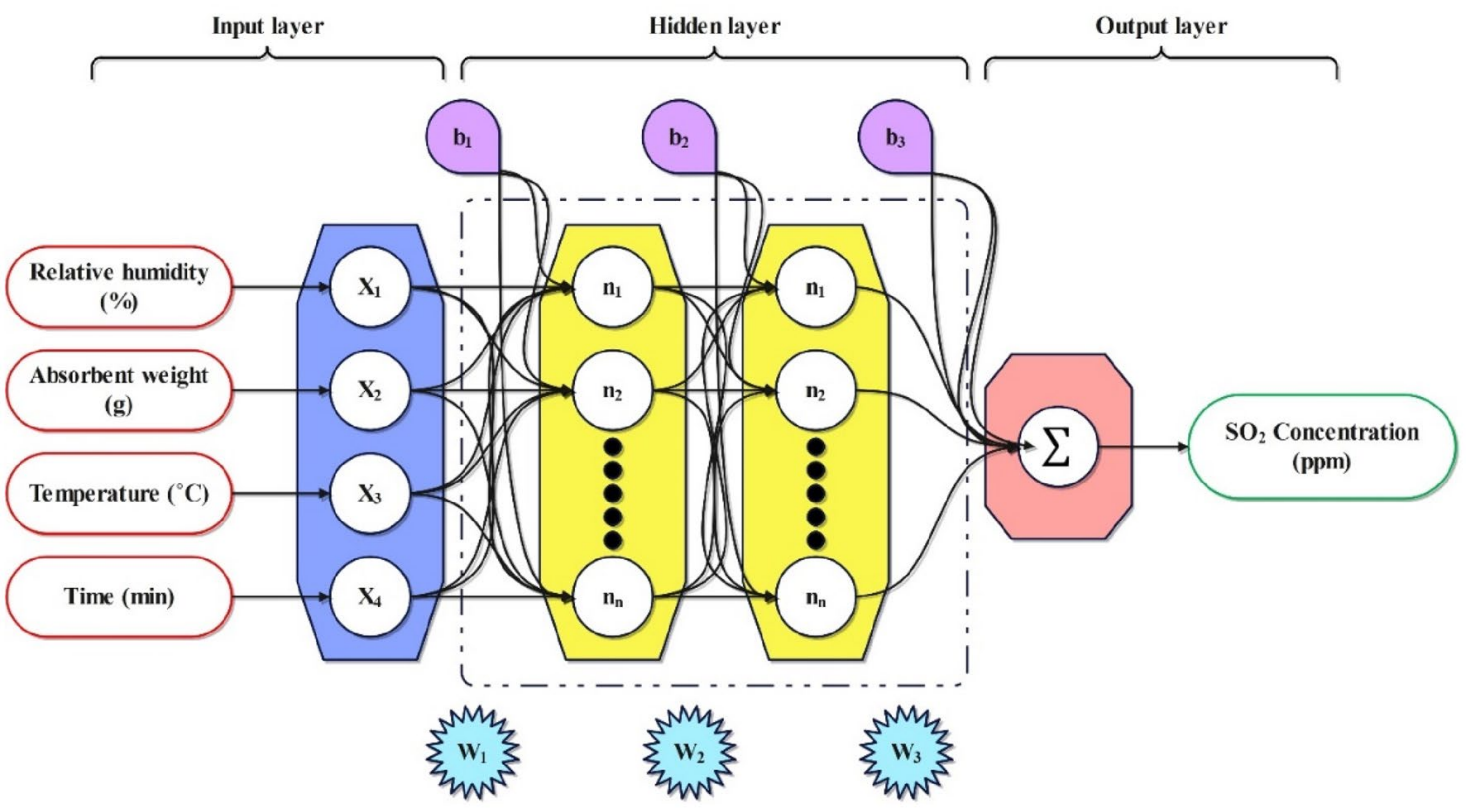
The architecture of the MLP model.

The perceptron algorithm, initially proposed by Rosenblatt in the late 1950s, has gained significant recognition as a prevalent and regularly utilized model in supervised ML^34^. Compared to more intricate models, the MLP offers higher model quality, simplicity of implementation, and shorter training duration^35^. In the MLP network, the input layer receives information and transmits it to the output layer, reflecting the final findings. Meanwhile, the hidden layers within the network do the initial processing of the received data. The hidden layers of the neural network receive the weights and biases and subsequently propagate the values to the output layer through the utilization of activation functions^36^. Figure 5 illustrates the primary architecture of the MLP. The Eq. (4) comes from the MLP feature approach. In this equation, the output vector is denoted as g, the weight vector of factors is given by w, x_i_^k^ indicates the reference vector, and θ denotes the threshold limit^37^.

4 $$g=f(w{x}_{i}^{k}+\theta )$$

The output of the MLP neural network can be derived in the following manner:

5 $${\gamma }_{jk}={F}_{k}\left(\sum_{i=1}^{{N}_{K-1}}{w}_{ij}{\gamma }_{i(k-1)}+{\beta }_{jk}\right)$$

where γ_jk_ stands for the influence exerted by neuron j in layer k, while β_jk_ signifies the bias weight associated with neuron j within layer k. The term F_k_ denotes the nonlinear activation transfer function about layer k, and w_ij_ represents the connection weights.

#### Radial basis function neural network

**Figure 6:**
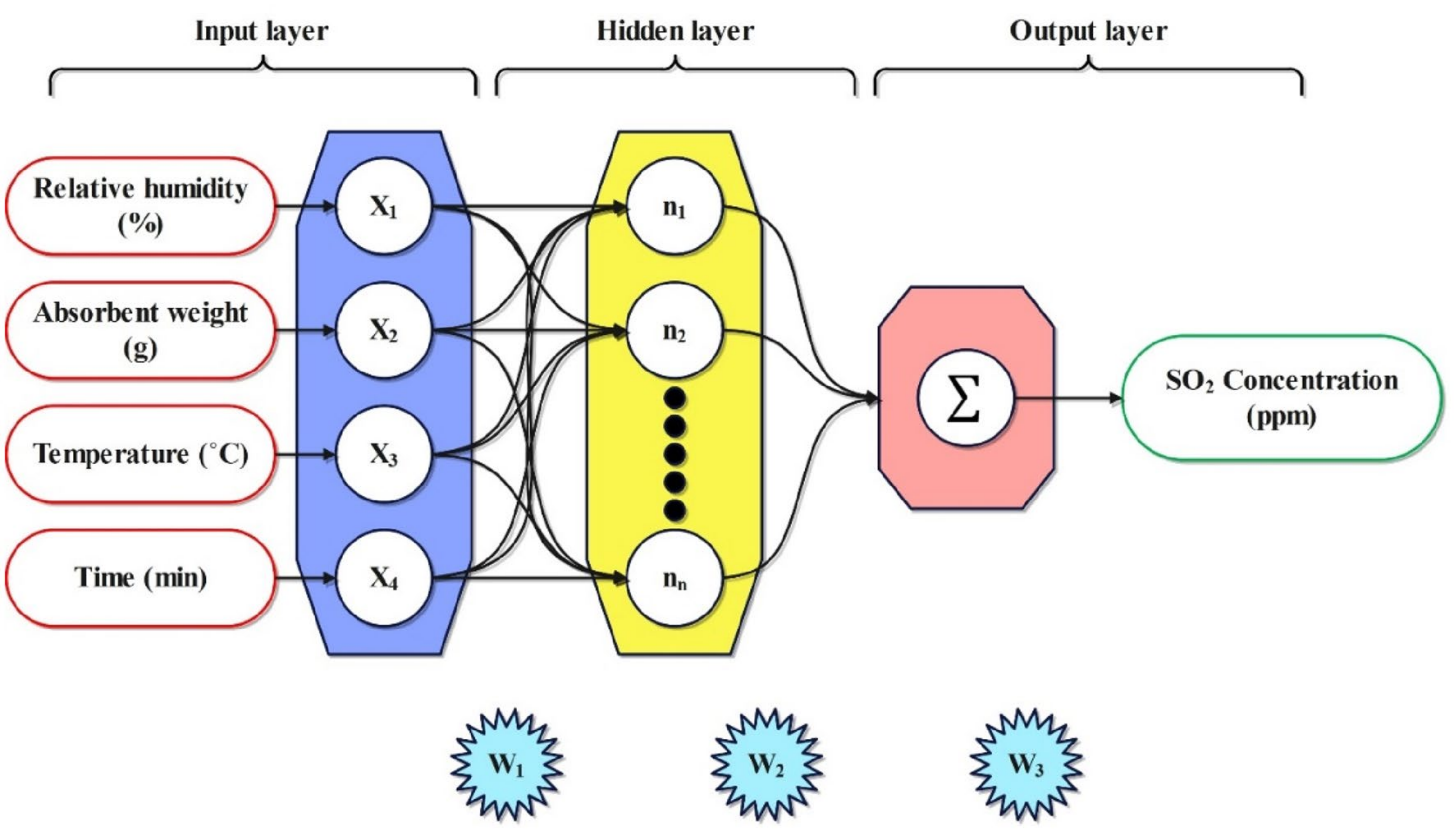
The architecture of the RBFNN model.

The RBFNN possess a robust mathematical basis deeply based on regularization theory, which is employed to address ill-conditioned problems^38^. The RBFNN model's versatility stems from its outstanding efficiency, simplicity, and speed, making it suitable for various applications^39^. An RBFNN is structured with three distinct layers: the input, hidden, and output layers. Each layer is assigned distinct tasks^40^. The transfer function within RBFNN exhibits nonlinearity when mapping inputs to hidden layers, but it demonstrates linearity when mapping hidden layers to output layers^41^. Equation (6) displays the Gaussian transfer function used by the RBFNN for processing inputs^42^.

6 $$G\left(\Vert x-{c}_{i}\Vert *b\right)=exp\left(-\frac{1}{2{\sigma }_{i}^{2}}{(\Vert x-{c}_{i}\Vert *b)}^{2}\right)$$

where the input variable is denoted as x, the center point is represented by c_i_, the bias is symbolized as b, and the spread of the Gaussian function is indicated by σ_i_. Figure 6 illustrates an essential schematic representation of the RBFNN.

#### Random forest

**Figure 7:**
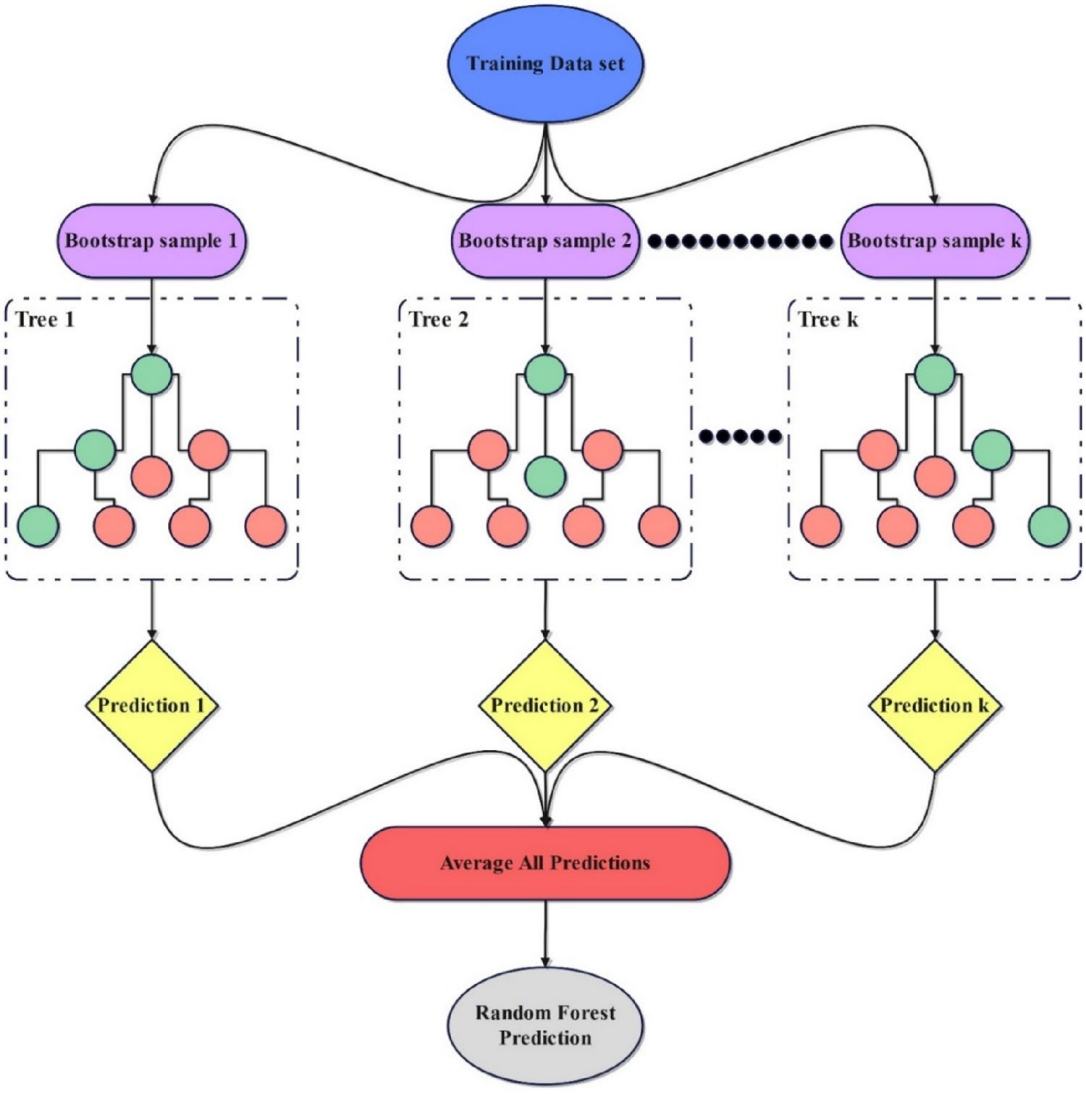
Schematic diagram of RF procedure.

The RF algorithm is widely recognized in the field of ML for its ability to construct predictive models, and it was initially proposed by Breiman^43^ in 2001. This supervised learning technique is a composite model consisting of several tree predictors. Each tree predictor is constructed based on the values of an independent random vector, and all vectors are created with the same configuration. This method is applicable for solving classification and regression issues^44,45^. The functioning of the RF model is depicted in Fig. 7. Each regression tree’s output was added together to get the result shown in Eq. (7) below^46^:

7 $$R\left(x\right)=\frac{1}{K}\sum_{i=1}^{K}{T}_{i}(x)$$

where T_i_(x), x, and K represent an individual regression tree that is constructed using a subset of input variables and bootstrapped samples, a vector input variable, and the number of trees, respectively.

RF can assess the significance of input features, improving model's performance when dealing with datasets with many dimensions. The process entails quantifying the average reduction in predictive accuracy resulting from altering a single input variable while holding all other variables constant. This process entails assigning a score that represents the relative relevance of each variable, which then aids in selecting the most impactful features for the ultimate model^47^.

#### Extra trees regression

Geurts et al.^48^ proposed the ETR method, a developed method derived from the RF model. This approach is a recent advancement in ML, an enlargement of the well-known RF algorithm. It was made to prevent overfitting. Training each base estimator with a random subset of features is fundamental to the ETR algorithm's success, just as in the RF^47^. ETR uses the whole training dataset to train each regression tree. On the other hand, RF uses a bootstrap replica to train the model^49^.

#### Support vector machine

Previously, supervised learning approaches, specifically SVM, were mainly utilized for classification purposes. However, contemporary research has also demonstrated successful adaptations of these techniques for regression problems^50^. Furthermore, kernel functions are employed in SVM to transform the training data, thereby mapping it to a space with higher dimensions where the data can be effectively segregated^51^. SVM models were built using consistent input descriptors and training/testing datasets. Equation (8) within the SVM model is the prediction or approximation function^52^.

8 $$f\left(x\right)=\sum_{i=1}^{l}{\alpha }_{i}K(x,{x}_{i})+b$$

**Figure 8:**
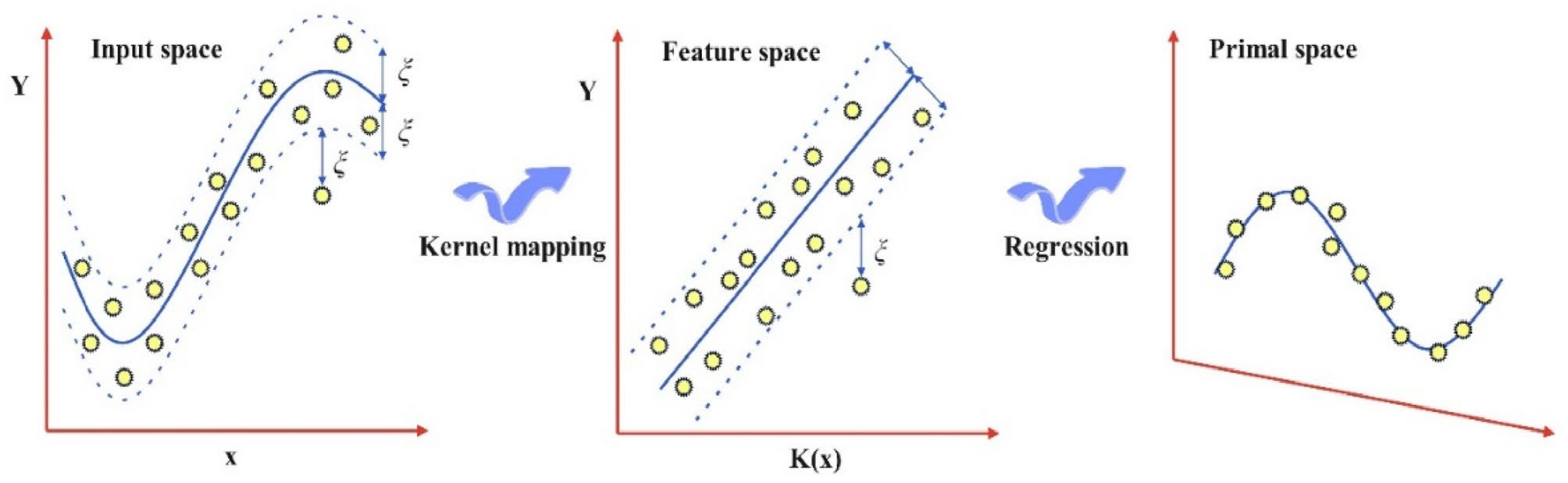
The main structure of the SVR.

SVM helps minimize systemic risk, diminishing overfitting, lowering prediction errors, and enhancing generalization. SVM does not rely on a predefined structure since it assesses the significance of training samples to determine their contributions. "Support vectors" are only established for models based on specific data samples^53^. In this research, SVM regression was conducted using the support vector regression (SVR) class available in the scikit-learn API's SVM module. As illustrated in Fig. 8, a model is crafted, and the data is transformed into a chosen dimension.

### Error metric

The models are evaluated based on several metrics, including mean absolute error (MAE), mean squared error (MSE), root mean squared error (RMSE), and regression coefficient (R^2^), to choose the optimal model. The MAE is calculated as the average of the absolute values of the errors. The metric is defined as the arithmetic mean of the absolute differences between the actual values and the corresponding predicted values. The term "MAE" is commonly used to denote a loss function. The primary objective in utilizing this loss function is to minimize it. The definition of MAE is as follows^54^:

9 $$MAE=\frac{1}{n}\sum_{i=1}^{n}\left({Y}_{predicted}-{Y}_{actual}\right)$$

where Y_predicted_ indicates the predicted value, and Y_actual_ represents the actual value of the model.

The MSE denotes the average value of the squared error, as illustrated in Eq. (10). MSE is seen as a loss function that requires minimization. One of the primary rationales for the extensive utilization of MSE in practical ML applications stems from its inherent characteristic of assigning more penalties to more significant errors compared to MAE when employed as the objective function^54^.

10 $$MSE=\frac{1}{n}\sum_{i=1}^{n}{\left({Y}_{predicted}-{Y}_{actual}\right)}^{2}$$

The RMSE is mathematically defined as the square root of the MSE, as demonstrated in the equation below. The RMSE is widely utilized as a loss function due to its interpretative capacity^54^.

11 $$RMSE=\sqrt{\frac{1}{n}\sum_{i=1}^{n}{\left({Y}_{predicted}-{Y}_{actual}\right)}^{2}}$$

The coefficient of determination (R^2^) is a way to measure how well the model fits the scientifically reliable results. The better the estimates are based on the experimental data, the closer the R^2^ is to 1. The calculation for R^2^ is as follows^55^:

12 $${R}^{2}=\frac{\sum_{i=1}^{n}{\left({Y}_{predicted}-{Y}_{actual}\right)}^{2} }{\sum_{i=1}^{n}{\left({Y}_{predicted}-{Y}_{mean}\right)}^{2}}$$

where *Y*_*mean*_ refers to the average value.

## Results and discussion

In this study, Kaggle's CPU session was employed, offering an environment equipped with 4 CPUs. The specifications of these CPUs include an Intel(R) Xeon(R) CPU @ 2.20 GHz with a total of 4 CPU cores, supporting both 32-bit and 64-bit operations. Dedicating 1 CPU to each trial facilitated the concurrent execution of 4 processes, streamlining the exploration of hyperparameter space for each model. The duration of hyperparameter tuning for individual models spanned from 2 to 3 h, reflecting variations influenced by the intricacies of different models and the extent of the hyperparameter search space. During the hyperparameter tuning and model training phases, approximately 3-4 GB of RAM was employed. This allocation proved sufficient to manage the computational load throughout these processes.

### Hyperparameters optimization

In the ML domain, the crucial role of hyperparameter optimization in developing efficient and precise models is undeniable. The main objective is to fine-tune each model, ensuring optimal performance across diverse datasets. A cohesive strategy for hyperparameter tuning was adopted, utilizing Ray Tune and various schedulers. The primary focus was to strike a balance between a model's complexity and its predictive accuracy, achieved through meticulous exploration and validation processes. This approach aimed to prevent overfitting and maintain the model's generalization ability. In the tuning process, practices like K-fold cross validation, early stopping, and L2 regularization played a pivotal role, especially for models such as ANN, MLP, and RBFNN. These practices effectively validated the model's performance and mitigated overfitting risks. Ray Tune's ASHAScheduler dynamically adjusted hyperparameters during training across various models, including ANN, RBFNN, RF, ETR, and SVR. The HyperBandScheduler was particularly effective for the MLP model, accelerating the tuning process and ensuring swift convergence to the best hyperparameter configuration. It is worth noting that other methodologies such as multi-objective optimization in neural architecture search (NAS) with algorithms like NSGA-II and the utilization of surrogate models for SVR are recognized as valuable tools that complement and enhance optimization strategies^56–58^.

#### ANN

After considering various factors such as the number of layers, neurons per layer, batch_size, learning_rate, weight_decay, activation_function, optimizer, and epochs, a thorough analysis was conducted to determine the best configuration for the ANN network architecture. The main goal of this analysis was to achieve the most favorable results on the test data. The optimal hyperparameters for the ANN network can be summarized as follows: units_layer1 = 128, units_layer2 = 128, units_layer3 = 32, batch_size = 16, learning_rate = 0.0005, weight_decay = 0.00002, activation_function = Relu, optimizer = Adam, and epochs = 216.

#### MLP

The optimal configuration of the MLP network architecture was determined by considering several factors, including the number of layers, the number of neurons for each layer, dropout, weight_decay, learning_rate, batch_size, test_size, activation_function, optimizer, and the number of epochs. This comprehensive analysis aimed to produce the most favorable outcomes on the test data. The ideal hyperparameters for the MLP network are summed up as follows: units_input = 256, units_hidden = 32, num_layers = 5, dropout = 0.0491, weight_decay = 0.00008, learning_rate = 0.0003, batch_size = 32, test_size = 0.2, activation_function = Relu, optimizer = Adam, and epochs = 100.

### RBFNN

**Figure 9:**
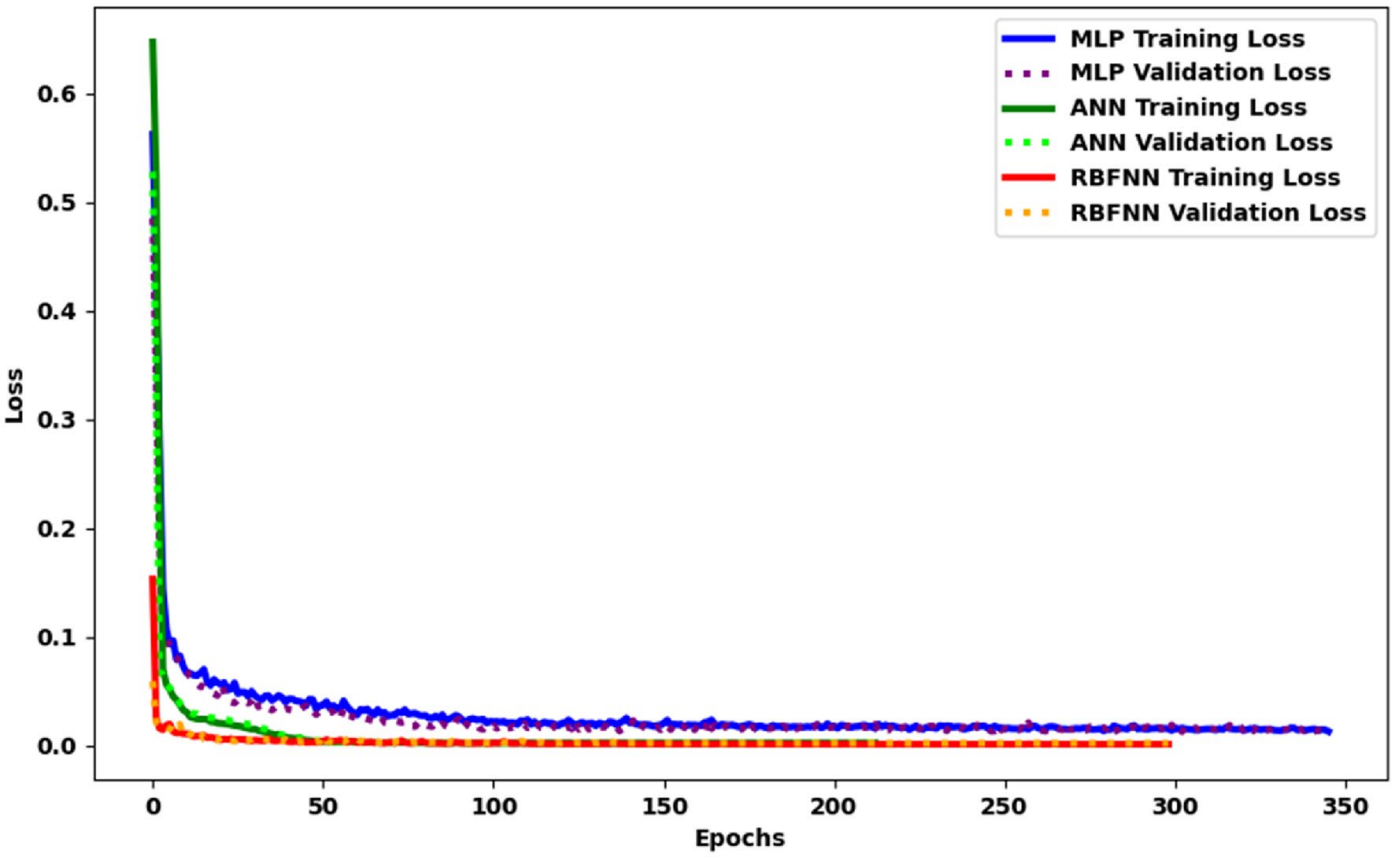
The learning curve of MLP, ANN, and RBFNN models.

The training of the RBFNN involves optimizing many network characteristics, including the number of epochs, hidden_features, weight_decay, learning_rate, activation_function, and optimizer to attain optimal performance on the test data. The optimized hyperparameters include the following values: the number of epochs = 1500, the hidden_features = 50, the weight_decay = 0.00000001, learning_rate = 0.1, activation_function = Relu, and optimizer = Adam. Figure 9 illustrates the learning curve according to the most influential architecture of the MLP, ANN, and RBFNN.

### RF

To enhance the performance of the RF algorithm, it is necessary to select appropriate hyperparameters carefully. The hyperparameters typically considered for optimization include n_estimators, max_depth, bootstrap, max_features, min_samples_leaf, criterion, and min_samples_split. For the specific case at hand, the ideal values for these hyperparameters are determined to be 74, 41, false, sqrt, 1, absolute_error, and 3 respectively, for n_estimators, max_depth, bootstrap, max_features, min_samples_leaf, criterion, and min_samples_split.

### ETR

To optimize ETR, these hyperparameters are assessed: (n_estimators, max_depth, min_samples_leaf, bootstrap, max_features, min_samples_leaf, criterion, and min_samples_split), the ideal values are n_estimators = 70, max_depth = 12, bootstrap = false, max_features = log 2, min_samples_leaf = 1, criterion = poisson, and min_samples_split = 5.

### SVR

The hyperparameters typically considered during the optimization of SVR include kernel, C, degree, gamma, coef0, epsilon, shrinking, and tol. In this case, the ideal values for these hyperparameters are as follows: kernel = RBF, C = 99.5403, degree = 3, gamma = scale, coef0 = 0.8938, epsilon = 0.0589, shrinking = true, and tol = 0.0014.

### Comparison predictions

**Figure 10:**
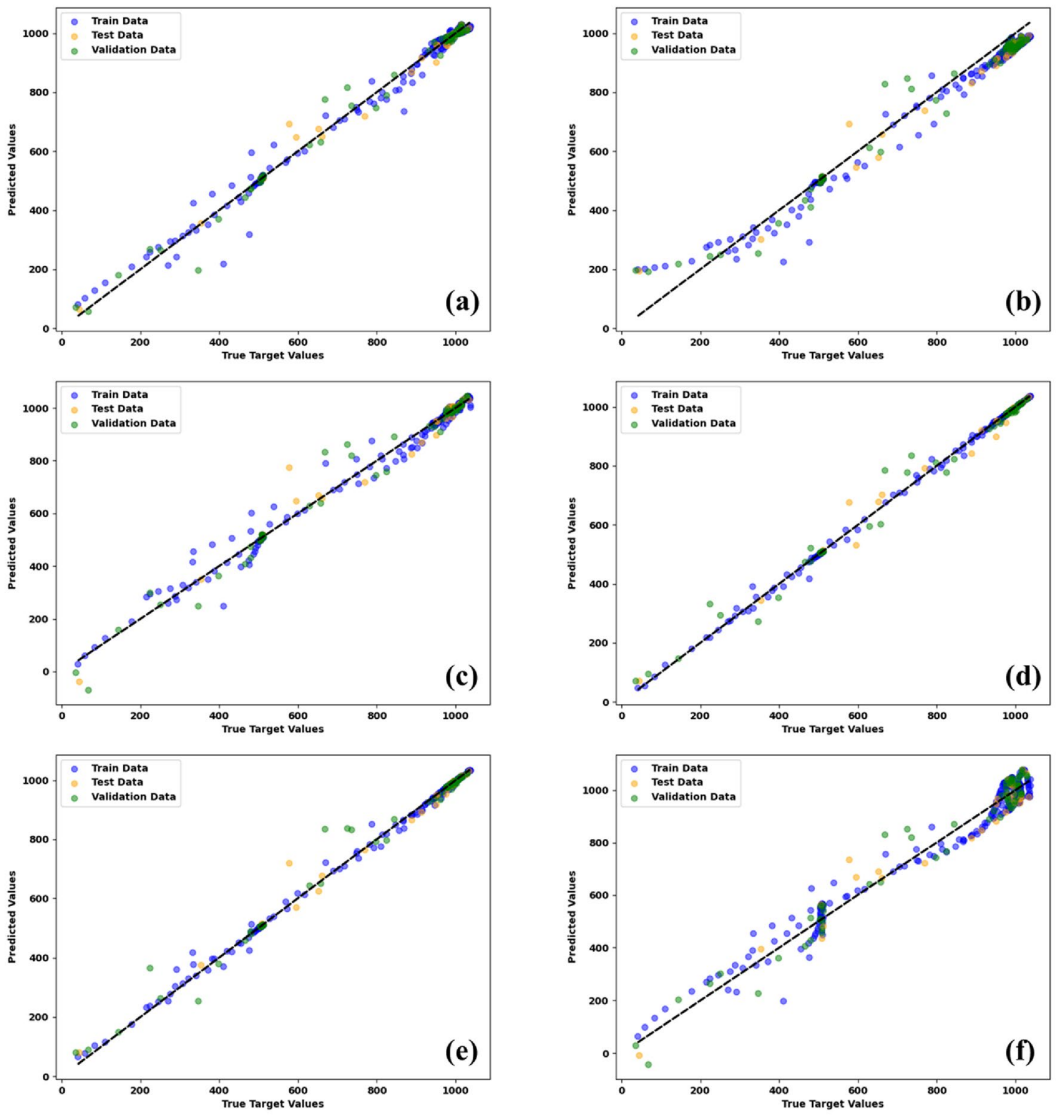
SO_2_ output concentration experimental versus predicted data using the models: (**a**) ANN, (**b**) MLP, (**c**) RBFNN, (**d**) RF, (**e**) ETR, and (**f**) SVR.

The models were retrained using the specified hyperparameters on training (70%), validation (20%), and testing (10%) datasets for each case. Following guidelines like those described in^59^, we constructed the testing dataset to ensure uniform coverage across the entire operational domain. This was achieved by systematically sampling points across the full range of each variable, including relative humidity, absorbent weight, temperature, time, and SO_2_ concentration. The graph in Fig. 10 compares the estimated SO_2_ concentration with the experimental values of the test groups. The performance of the models was evaluated using analytical criteria, namely the MAE, MSE, RMSE, and R^2^, as indicated in the previous equations. The outcomes are presented in Table 3. The high R^2^ value of 0.9902 and low MSE value of 0.0008 indicate that the RF model is suitable for estimating SO_2_ absorption by calcium silicate based on operational and absorption conditions. The model’s performance over the uniformly sampled testing dataset, which encapsulates the entire domain of FGD conditions, yielded a consistent accuracy, demonstrating its robustness and reliability in various operational scenarios. This precise ML model can predict the SO_2_ concentration under different operational conditions for new absorbents. The ML models developed in this study can reduce the time and cost associated with experimental screening tests for various absorbents used in different scenarios, thereby promoting cost-effective and environmentally friendly generation for sustainability. Figure 10 demonstrates a high level of accuracy in the relationship between the RF model outputs and the SO_2_ concentration data. The RF model achieves the most accurate results, accurately estimating the experimental data.

**Table 3:** Analytical criteria for comparing different models.

Model	R^2^			MAE			MSE			RMSE		
	Train	Validation	Test	Train	Validation	Test	Train	Validation	${\text{Test}}$	Train	Validation	Test
ANN	0.9884	0.9896	0.9878	0.0157	0.0166	0.0183	0.0008	0.0007	0.0010	0.0279	0.0270	0.0322
MLP	0.9704	0.9646	0.9594	0.0359	0.0410	0.0422	0.0020	0.0028	0.0028	0.0449	0.0530	0.0527
RBFNN	0.9878	0.9781	0.9674	0.0151	0.0215	0.0229	0.0008	0.0017	0.0019	0.0289	0.0415	0.0434
RF	0.9989	0.9869	0.9902	0.0032	0.0149	0.0145	0.00007	0.0009	0.0008	0.0083	0.0299	0.0279
ETR	0.9975	0.9835	0.9852	0.0057	0.0145	0.0146	0.0002	0.0011	0.0012	0.0130	0.0337	0.0342
SVR	0.9678	0.9555	0.9751	0.0392	0.0450	0.0381	0.0022	0.0030	0.0020	0.0469	0.0553	0.0445

**Figure 11:**
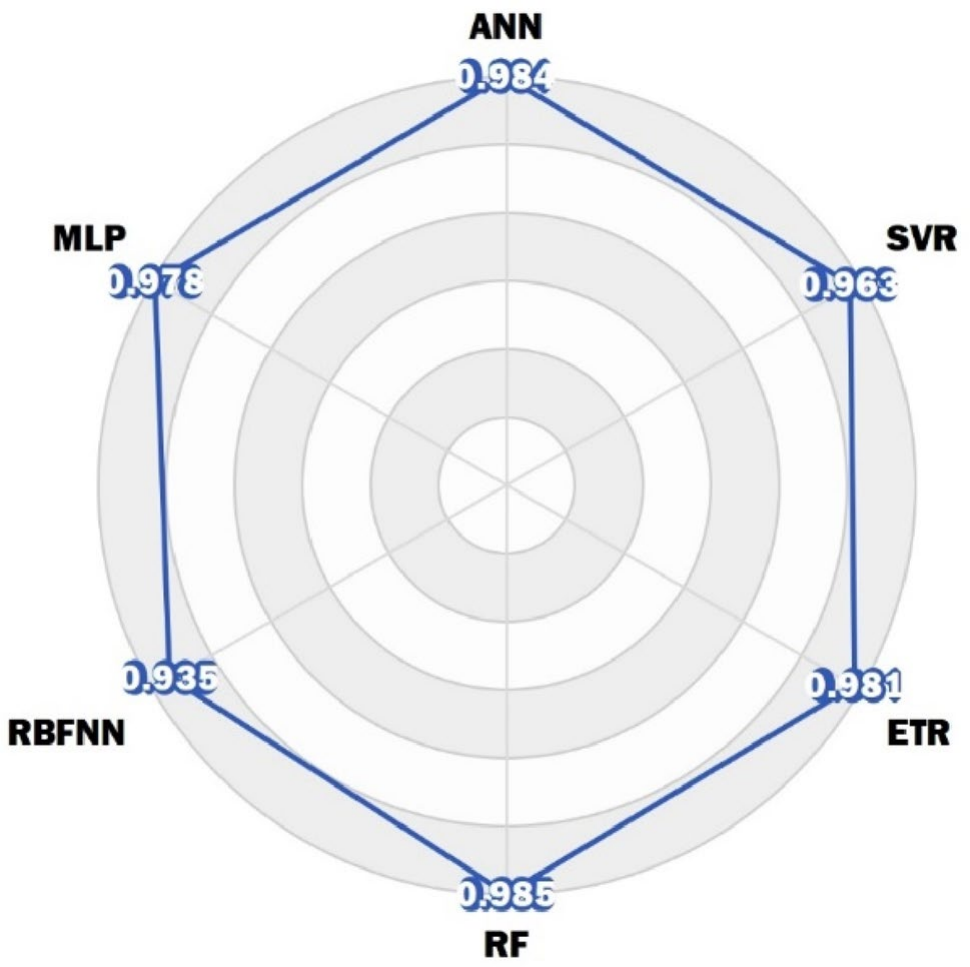
Radar chart showing the performance of models based on R^2^ value.

**Figure 12:**
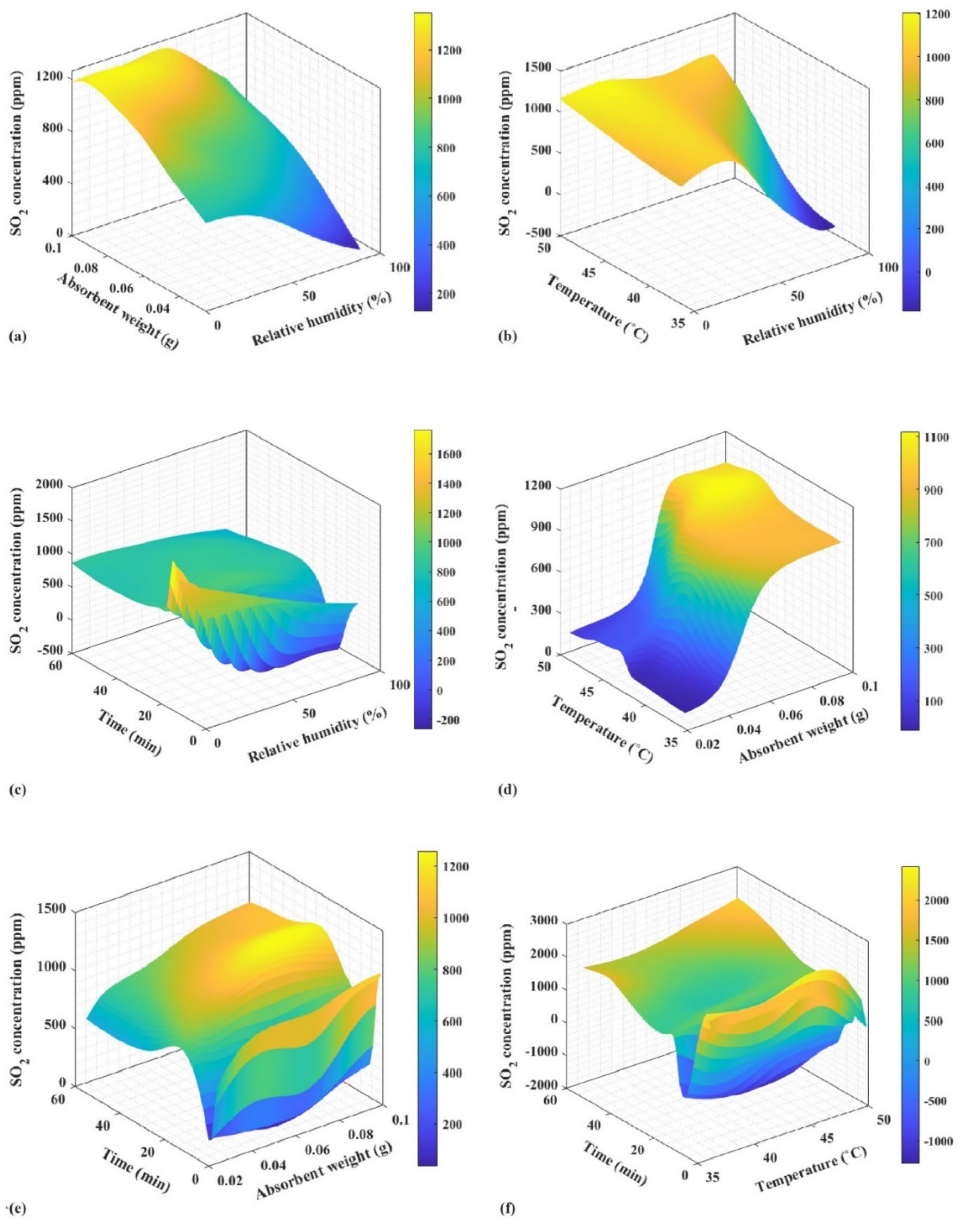
3D surface plots generated by the RF model to provide SO_2_ concentration performance for analyzing the influence of (**a**) relative humidity and absorbent weight, (**b**) relative humidity and temperature, (**c**) relative humidity and time, (**d**) absorbent weight and temperature, (**e**) absorbent weight and time, and (**f**) temperature and time.

A random selection of five test data points was made from the set of considered data to assess the validity of the acquired models. The data shown in Table 4 provides information on the experimental concentration of SO_2_. The calculated value is determined based on the specific operating conditions for each model. Furthermore, the RF model had the highest level of accuracy in predicting SO_2_ concentration across most cases, surpassing all other models. Figure 11 shows a radar chart to compare the R^2^ value of the models. Based on the data given, it can be concluded that the RF algorithm has superior performance in predicting experimental data about SO_2_ concentration. The training algorithm of the network aims to minimize the average error. Therefore, the RF model was employed to generate three-dimensional graphs that illustrate the correlation between input parameters or operational circumstances and the concentration of SO_2_. Figure 12 depicts the three-dimensional curves of the RF forecasting model. The collection of data on the curves was conducted to enhance comprehension of the impact of relative humidity, absorbent weight, temperature, and time on the concentration of SO_2_. The values of the constant parameters are determined by averaging the remaining inputs. A generalized optimal RF model to provide SO_2_ concentration performance for analyzing the influence of (a) relative humidity and absorbent weight; (b) relative humidity and temperature; (c) relative humidity and time; (d) absorbent weight and temperature; (e) absorbent weight and time; and (f) temperature and time, while other parameters are kept constant at 44% relative humidity, 0.0625 g absorbent weight, 43.25°C temperature, and 30.5 min time. Depending on the data presented in Fig. 12, maintaining the process at a higher relative humidity leads to a decrease in SO_2_ concentration. While humidity typically promotes the dissolution of SO_2_, it can also influence its concentration in the gas phase. High relative humidity can lead to increased water content in the flue gas, which, in turn, enhances SO_2_ absorption and decreases its concentration in the gas phase^60,61^. With the increase in the weight of the absorbent and with the increase of time, the concentration of SO_2_ increases significantly. This depends on various factors. Initially, increasing absorbent weight enhances SO_2_ absorption by providing more surface area for interaction. However, when saturation is reached, excess absorbent can hinder absorption, potentially leading to increased SO_2_ concentration. also, SO_2_ absorption can reach a chemical equilibrium. Adding absorbent weight might shift this equilibrium towards desorption, resulting in higher SO_2_ concentrations, especially when excess absorbent prevents an absorption-favorable equilibrium. On the other hand, the rate of SO_2_ absorption depends on surface area and chemical reaction kinetics. Increased absorbent weight can alter reaction kinetics, potentially slowing absorption and causing higher SO_2_ concentrations. Over time, absorbed SO_2_ can desorb back into the gas phase, increasing SO_2_ concentration, particularly with prolonged exposure^62–64^. The optimal range of absorbent weight to keep the SO_2_ concentration low is 0.025–0.06 g. As the desulfurization process begins, SO_2_ concentration increases. After the initial rise, around the 5-min mark, SO_2_ concentration reaches a minimum. This phase represents efficient SO_2_ removal from the gas phase as the absorbent starts absorbing SO_2_. Following the minimum concentration, SO_2_ concentration starts to rise again. This is due to factors like absorbent saturation or changes in the equilibrium between gas and absorbent. Towards the end of the time interval, SO_2_ concentration stabilizes and reaches an equilibrium. This equilibrium reflects a balance between continued SO_2_ release and absorption by the absorbent^65–67^. The performance of SO_2_ concentration was insensitive to temperature changes.

**Table 4:** Calculation of FGD parameters by models by fitting the experimental data. Significant values are in bold.

Run	Relative humidity	Absorbent weight	Time	Actual value	ANN	MLP	RBFNN	RF	ETR	SVR
1	29	0.100	16.0	**1007.3**	1003.8	1013	1017.5	1004.2	1012.9	1027.4
2	58	0.100	8.5	**526.5**	564.7	556.5	545.3	534.1	566.3	601.8
3	0	0.100	30.0	**1012.2**	1019.5	1018.3	1002.9	1008.2	1007	1037.7
4	88	0.100	18.5	**813.3**	763.1	825.9	831.1	808.6	770.3	768.9
5	58	0.025	8.0	**454.0**	431.1	463.8	481.5	466.2	430.4	437.8

### Global sensitivity analysis (GSA)

**Figure 13:**
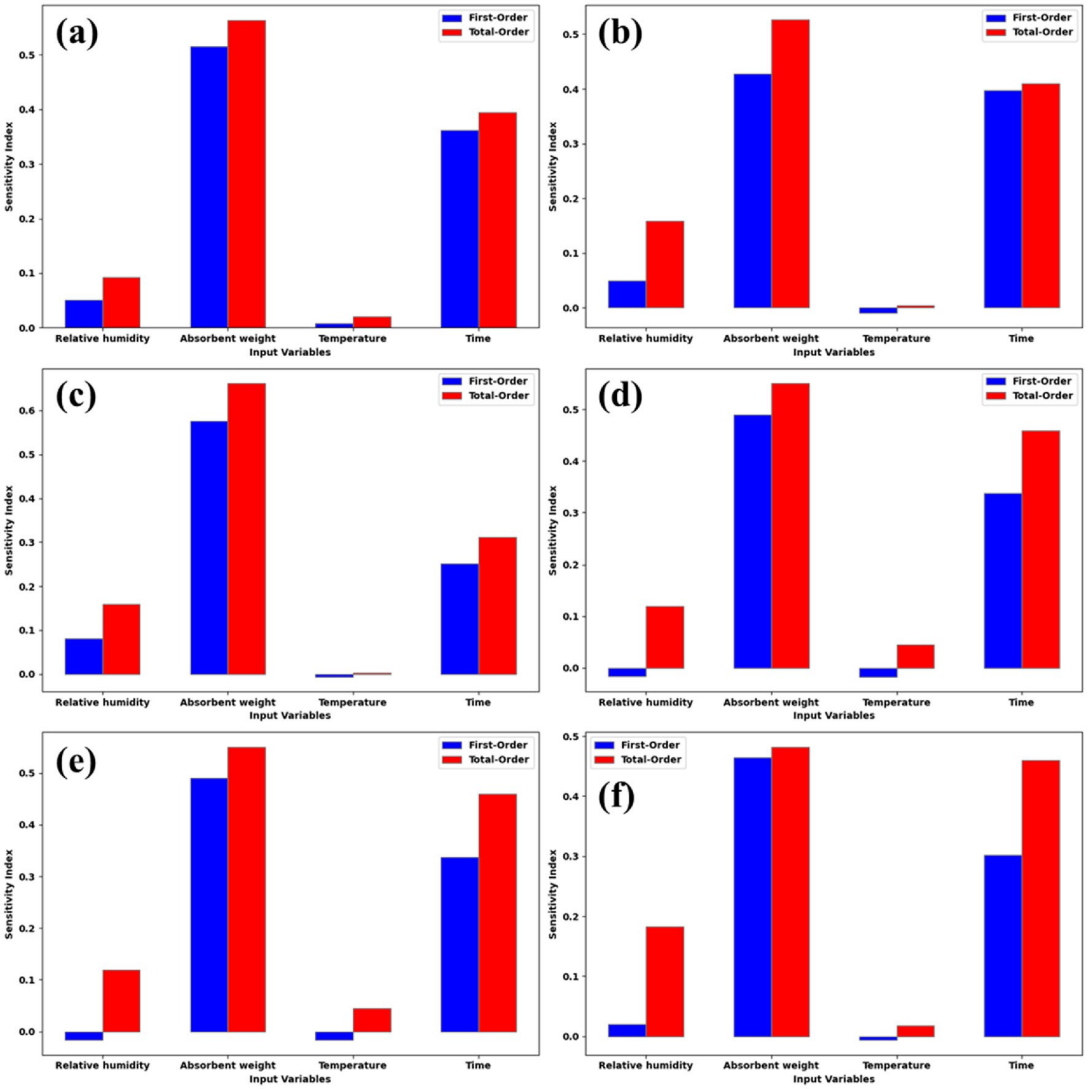
First order and total sensitivity indices in GSA using the models: (**a**) RBFNN, (**b**) ETR, (**c**) RF, (**d**) SVR, (**e**) MLP, and (**f**) ANN.

To identify the primary factors influencing the SO_2_ concentration, we conduct global sensitivity analysis (GSA) utilizing the ML models we developed. In this process, we apply the sensitivity equations provided in reference^68^. The GSA outcomes, specifically the first-order and total-order indices, are presented in Fig. 13 for the ANN, MLP, RBFNN, RF, ETR, and SVR models, respectively. The first-order index gauges the impact of individual environmental parameters on the output in isolation. Conversely, total order indices measure the influence of an environmental parameter, considering its interactions with other environmental factors^69^. Due to the computational complexity associated with determining higher-order indices individually, the calculation of total-order indices is commonly carried out. In all GSA simulations, we utilized 256 samples to assess the impact of each input parameter on the output. As depicted in Fig. 13, the output of all six models is most significantly influenced by the quantities of absorbent weight and time. Specifically, in the RBFNN and ETR models, time and absorbent weight respectively exhibit the foremost impact on the SO_2_ concentration. Conversely, in the RF, SVR, MLP, and ANN models, the absorbent weight and time respectively exert the greatest influence on the SO_2_ concentration. It is noteworthy that the impact of relative humidity and temperature on the SO_2_ concentration in all six models is deemed insignificant.

## Conclusion

This research studied calcium silicate absorbent to establish an ML prediction for SO_2_ concentration in an FGD process. The experimental data, which included 323 data sets, was defined with four inputs: relative humidity, absorbent weight, temperature, and time, and one output, including SO_2_ concentration. Six models were created to estimate the output parameters, including ANN, MLP, RBFNN, RF, ETR, and SVR. For the models mentioned earlier, statistical values such as the R^2^ and MSE were determined to determine the optimal model and evaluate the fitting effectiveness. The highest performance was provided by the RF model that demonstrated the best estimation with R^2^ of 0.9902 and MSE of 0.0008, and the optimal hyperparameter values were established as follows: n_estimators = 74, max_depth = 41, bootstrap = false, max_features = sqrt, min_samples_leaf = 2, criterion = absolute_error, and min_samples_split = 3. The predicted SO_2_ concentration closely matched the experimental results, demonstrating the accuracy of the modeling. Three-dimensional surface plots were reported to investigate the effect of relative humidity, absorbent weight, temperature, and time on SO_2_ concentration. The findings revealed that absorbent weight and time were the most influential factors in SO_2_ concentration among the four parameters investigated. The results of this investigation indicate that ML methods can significantly improve the prediction of SO_2_ concentration within the range of the experiment. Continued research and development in this field and advances in ML techniques hold great potential for achieving cleaner air quality, reduced environmental impact, and more efficient energy production through enhanced FGD processes. We hope this study contributes to the ongoing efforts to address environmental challenges and promote cleaner, more sustainable industrial practices.

### Supplementary Information

Supplementary Information.

## Data Availability

The datasets used and analyzed during the current study are available from the corresponding author upon reasonable request.

## List of symbols

${\alpha }_{i}:$: Weight for feature vector
b: Bias (–)
c_i_: Center points (–)
f: Function
F_k_: Nonlinear activation transfer functions
g: Output vector (–)
G: Gaussian function
i: Subscripts refer to the initial condition
i: Number of neurons in the hidden layer
k: Position vector
K: Kernel function
K: Number of trees
n: Number of neurons
N: Number of datasets for training (–)
R^2^: Coefficient of determination
t: Time (min)
T_i_: The result from each tree
w: Weight factor (–)
W: Absorbent weight
W_ij_: Weight related to each hidden neuron (–)
x: Input variable (–)
x_i_: The ith feature vector (–)
x_i_^k^: Reference vector (–)

## Greek letters

${\beta }_{jk}:$: Bias weight for neuron j in layer k
${\gamma }_{jk}:$: Neuron j’s output from k’s layer
$$\uptheta :$$: Threshold limit (–)
ξ: Slack variable
σ: Width of Radial Basis Function Neural Network (RBFNN) kernel (–)
σ_i_: Spread of Gaussian function (–)

## Abbreviations

ANN: Artificial neural network
CFF: Cascaded forward neural network
CNN: Convolutional neural networks
DFGD: Dry flue gas desulfurization
DNN: Deep neural network
ELM: Extreme machine learning
ETR: Extra trees regression
FGD: Flue gas desulfurization
GSA: Global sensitivity analysis
LSTM: Long short-term memory
LSSVM: Least squared support vector machine
MAE: Mean absolute error
MAPE: Mean absolute percentage error
ML: Machine learning
MLP: Multilayered perceptron
MSE: Mean squared error
NAS: Neural architecture search
RBFNN: Radial basis function neural network
RF: Random forest
RMSE: Root mean square error
RNN: Recurrent neural network
STD: Standard deviation
SVR: Support vector regression
WFGD: Wet flue gas desulfurization

## Terminology

Activation function: The activation function is a mathematical function between the input feeding the current neuron and its output going to the next layer
Bias: Bias is a constant that helps the model in a way that can fit best for the given data
Epoch: In the training process, the inputs enter each training step and give outputs compared with the target to calculate an error. With this process, weights and biases are calculated and modified in each epoch
Neurons: Neurons are the basic units of the large neural network
Weight: Represents the importance and strengths of the feature/input to the Neurons
